# The need for a rationalist turn in evidence‐based medicine

**DOI:** 10.1111/jep.12974

**Published:** 2018-06-28

**Authors:** Michael P. Kelly

**Affiliations:** ^1^ Department of Public Health and Primary Care, Institute of Public Health University of Cambridge Forvie Site Cambridge CB2 0SR UK

**Keywords:** clinical guidelines, Cochrane, epistemology, evidence‐based medicine, NICE, practical reasoning

## Abstract

When evidence‐based medicine (EBM) became established, its dominant rhetoric was empiricist, in spite of rationalist elements in its practice. Exploring some of the key statements about EBM down the years, the paper examines the tensions between empiricism and rationalism and argues for a rationalist turn in EBM to help to develop the next generation of scholarship in the field.

## 1 INTRODUCTION

In this paper, it is argued that evidence‐based medicine (EBM) is dominated by an empiricist rhetoric. The paper will explore the tension between that rhetoric and the rationalist elements of its practice. The history and the interaction between empiricist claims and underlying rationalist assumptions will be explored by examining the contributions of writers like Cochrane and Sackett and institutions like the Cochrane Collaboration and the National Institute of Clinical Excellence (NICE). The terms rationalism and empiricism are used here in Hume's sense of rationalism being about the relationship between ideas and empiricism being concerned with matters of fact.^1^ The importance of rationalism and empiricism in medicine in general and in EBM in particular has been noted by other commentators.^2^ The argument here is built on the Humean approach and on the older idea of phronesis as described for example by Montgomery.^3^ An empiricist approach is in and of itself is neither bad nor negative. However, this style of thinking has set the boundaries for what EBM does and does not do, particularly with respect to questions of inference, judgement, and method. This paper goes on to make the case for an explicit rationalist turn (rationalist again in Hume's sense). The purpose is to help rebalance the epistemologies of EBM, and to make explicit a number of ideas, which have been around since the very beginning, but which have seldom found their theoretical or methodological voice.

There are a number of landmarks in the development of EBM. The ones chosen to illustrate the argument here are Cochrane's critique of the medical profession, the appearance of the McMaster school and the influential writings of Sackett and his colleagues, the founding of the Cochrane Collaboration, and then the establishment of NICE. Archie Cochrane focussed on ways to determine effectiveness and cost‐effectiveness; the McMaster group were intent on applying the lessons of public health epidemiology to clinical medicine; the main thrust of the Cochrane Collaboration was the improvement in methods of review and scientific reporting, and NICE was about putting EBM into practice. All had an explicit focus on empiricist methods and a very strong rhetoric about objective science and matters of fact. Yet in the work of the McMaster group, the Cochrane Collaboration, and NICE, there was always a tension. Some problems simply could not be solved by empiricist solutions, and rationalism never really went away.

## 2 ARCHIE COCHRANE: EFFECTIVENESS AND EFFICIENCY 1972

The contribution of Cochrane's book *Effectiveness and Efficiency: Random Reflections on Health Services* 4 published in 1972 is important because so much of the way that EBM subsequently developed was influenced by Cochrane's ideas. Cochrane asked a series of questions about clinical medicine. The questions were as follows. Do we know whether intervention x for problem y is effective? How do we know it is effective? How do we know whether it is more or less effective than intervention z? On what basis do we make that judgement of effectiveness? Do we know what it costs? Is it cost effective? If it is not cost effective, why is it being used? What are the dangers posed to patients of treatments about which we are scientifically uncertain? Are interventions dangerous? Why do we use potentially dangerous or worthless interventions? He argued that evidence derived from properly conducted investigations was the best way to answer these questions.

Cochrane concluded that the optimal way to determine whether a drug worked and whether it was better or worse than usual treatment or some other drug was the evidence from randomized controlled trials—now of course the standard method, but not then. He also called for a system to monitor and systematically record trial results. Further, although he did not describe it as such, Cochrane provided a sociological analysis of the way the medical profession operated. Powerful medical schools and senior professors in them—in England in the highly prestigious London Teaching Hospitals for example—dominated pedagogy, curricula, teaching, and paradigmatic understandings of disease aetiology, diagnostics, and therapeutics. The paradigms inevitably lagged some way behind leading research discoveries and innovations. They still do.^5^ Cochrane suggested that many doctors stayed with the practices taught during their medical education rather than adopting new treatments as they became available. Cochrane saw the profession as inherently conservative, slow to innovate, and slower still to link clinical practice to research evidence. In short, Cochrane wanted to modernize the profession so that up‐to‐date scientific evidence, especially that drawn from clinical trials, was the basis of practice rather than habits and ideas that were well past their sell by dates.^6^

So in the questions that Cochrane asked, we see a strong emphasis on evidence of observable phenomena based on scientific rigour and method. Evidence should drive practice, not opinions and social networks. His ideas in other words were strongly empiricist (in Hume's sense). Of course, Cochrane was a very sophisticated thinker on a range of medical and scientific matters,^7^ and so, this is not to caricature a very important reformer. It is to suggest though that the emphasis he, rightly, put on the way to bring about change in the profession foregrounded empiricism and left rationalism out of the account. It was with the next generation of thinkers about evidence and medicine that the pre‐eminence of empiricism really crystallizes.

## 3 DAVID SACKETT AND THE MCMASTER SCHOOL

The story shifts to North America and to a group of doctors and scholars linked to David Sackett at McMaster University in Hamilton, Ontario. They coined the term EBM. Sackett's original idea was to apply the principles of public health epidemiology to clinical medicine.^8^ In 1992, the Evidence‐Based Medicine Working Group led by Sackett and his colleague Gordon Guyatt published a programmatic statement in which they argued that:

“Evidence‐based medicine de‐emphasizes intuition, unsystematic clinical experience, and pathophysiologic rationale as sufficient grounds for clinical decision making and stresses the examination of evidence from clinical research”.^9^

Note that from the outset, they acknowledged the role of clinical experience and judgement, but argued that on their own, these were not sufficient for effective medical practice and were a source of potential bias. They emphasized instead the importance of systematic, reproducible unbiased observations. The group argued that the understanding of basic mechanisms of disease is a necessary but not sufficient guide for clinical practice. Their contention was that:

“Understanding certain rules of evidence is necessary to correctly interpret literature on causation, prognosis, diagnostic tests, and treatment strategy. It follows that clinicians should regularly consult the original literature (and be able to critically appraise the methods and results sections) in solving clinical problems and providing optimal patient care. It also follows that clinicians must be ready to accept and live with uncertainty and to acknowledge that management decisions are often made in the face of relative ignorance of their true impact.” 9

They set out to put this into practice in their teaching at McMaster.^9^ It is important to note here their emphasis on *rules* of evidence. By this was meant procedures and protocols to make sense of evidence. Although the group acknowledged clinical experience and judgement, they did not seek to apply the idea of rules to these judgements and neither did they acknowledge that the application of rules is an interpretive process, itself requiring judgement.

In 1995, Sackett and Rosenberg outlined 5 principles of EBM. These were that clinical decisions should be based on the best patient, population, and laboratory evidence; that evidence rather than habits, protocols, or traditions should drive practice; that the best evidence integrated epidemiology, biostatistics, and pathophysiology, and techniques like meta‐analyses of randomized trials and economic analyses (not personal opinion) should be used to appraise that evidence; that evidence must inform practice; and that practice should be continuously evaluated.^10^ The echoes of Archie Cochrane are clear, as are the strongly empiricist assumptions about the superiority of observable empirical data over other forms of thinking.

In 1996 in a landmark and much quoted editorial, Sackett and colleagues provided what has become the standard definition of EBM as

“the conscientious, explicit, and judicious use of current best evidence in making decisions about the care of individual patients. The practice of evidence based medicine means integrating individual clinical expertise with the best available external clinical evidence from systematic research”.^11^

Sackett et al defined external evidence as:

“clinically relevant research, often from the basic sciences of medicine, but especially from patient centred clinical research into the accuracy and precision of diagnostic tests (including the clinical examination), the power of prognostic markers, and the efficacy and safety of therapeutic, rehabilitative, and preventive regimens … [EBM] involves tracking down the best external evidence with which to answer our clinical questions … Because the randomised trial, and especially the systematic review of several randomised trials, is so much more likely to inform us and so much less likely to mislead us, it has become the ‘gold standard’ for judging whether a treatment does more good than harm.”^11^

Again, it is important to reflect on the words “best” and “gold standard”. What is best and a gold standard are judgements, albeit judgements that can be assisted by certain rules and protocols, but they are still judgements.

This editorial was published against a background of growing opposition to EBM and not a little controversy. One objection was that EBM was nothing new and that doctors were already practicing EBM. Sackett and colleagues' riposte was that in medical practice, things had not changed since the publication of Cochrane's book 22 years before. The authors were keen to emphasize that best evidence did not replace the diagnostic or therapeutic skills of the physician. They were at pains to point out that the best evidence was a platform for better clinical judgement and that it would not in any sense replace it. They also noted that the rate of publication of new papers made it simply impossible for even the most conscientious physician to keep up with the literature, so to consult reviews of accumulated evidence was a *sine qua non* of EBM. The tone is conciliatory up to a point, acknowledging the role of experience and judgement, but the question of how experience and judgement integrate with the rules remains oblique.

So, the ideas from Sackett and colleagues did articulate a view in which observable and replicable evidence, properly reported and appraised, should be the cornerstone of practice. The methods that they advocated like the randomized controlled trial (RCT) and the focus on the elimination of bias in the relationship between independent and dependent variables were inherently reductionist. The notions of causation and association embedded in trial design are linear. The emphasis on the importance of evidence over opinion elevates science above other kinds of values in the same way that Cochrane had argued in his original book. At the same time, the thorny problem of clinical judgement and expertise and experience hovers round the margins of the discussion and never really goes away.

## 4 THE COCHRANE COLLABORATION

In 1993, the Cochrane Collaboration was established, taking its name from Archie Cochrane and his suggestion that there should be a system for making available systematic reviews of all relevant RCTs of health care. It was led initially by Iain Chalmers and took on the task, through a network of volunteers, of cataloguing and reviewing the extant trials and establishing them in an accessible data base.^12^, ^13^ It was supported at first by UK NHS R&D funding.^14^ The approach the Collaboration took had already been pioneered by Chalmers in his work on obstetric interventions carried out in the National Perinatal Epidemiology Unit in Oxford.^15^ The principle was that not only should trials themselves be conducted to the very highest standards, but so too should the review and appraisal and synthesis of the results of those trials. Chalmers was also very clear that the failure to publish results, especially negative ones, was a particular weakness in the evidence base.^16^ The thrust of the thinking was about objectivity, accurate measurement, honest reporting, and the importance of accumulating evidence to get towards more and more precise answers to clinical questions.

The existence of the Cochrane Collaboration and all the work it has done has been a major pillar in the development of EBM. It is a touchstone of content, techniques, and method, and it has helped to build the reputation of EBM. It has become a resource much used by researchers, guideline developers, and clinicians worldwide. It has generated an industry of methodologists exploring many of the finer details of process and procedure. The original idea that there should be a repository of trials and that through the process of accumulation, synthesis, and appraisal that repository can provide better answers to questions of efficacy and effectiveness remains at the heart of what those associated with Cochrane do.^17^, ^18^ The overarching principle is empiricist in the sense that the weight of argument comes from the volume of good empirical evidence and that very mass provides the clinching argument against anecdote, judgement, and habit. The weight of evidence will be the deciding factor, not politics or power struggles between competing groups of scientists or doctors. This is a very important aspiration and one to be encouraged. But as will be argued below, there is another, rationalist, side to the argument.

## 5 NICE

In 1999 in England, NICE was established to conduct appraisals of new drugs to determine their value for money for the NHS. It was an attempt to end the so‐called postcode lottery of health care in England and Wales, where treatments that were available depended upon the NHS Health Authority area in which the patient happened to live. Soon afterwards, NICE began developing clinical guidelines. In April 2005, NICE amalgamated with the Health Development Agency to become the new National Institute for Health and Clinical Excellence (still abbreviated as NICE) now with responsibility for developing public health guidelines. Following the Health and Social Care Act 2012, NICE was renamed the National Institute for Health and Care Excellence on April 1, 2013 (still with the acronym NICE) reflecting its then latest responsibilities for publishing social care guidelines.

The National Institute of Clinical Excellence is important in the story of EBM because more than any other public body in the UK, and a public body that found itself very much in the in the public eye, it executed and practiced the principles of EBM. It drew heavily on the thinking that had followed in the wake of the 1990s NHS R&D strategy.^14^ The National Institute of Clinical Excellence developed its own procedures to conduct its technology appraisals and to develop guidelines, but these leant heavily on the groundwork done both by pioneers in EBM as well as the health economics developed especially at the University of York.^19^

However, it was in the execution of its tasks that it became clear to a number of the leading figures at NICE, especially Sir Michael Rawlins the Chair, that there were underlying assumptions, which presented NICE's appraisal and guideline development committees with some very knotty problems that were not amenable to solutions derived from the empiricist precepts of EBM. The first efforts NICE made to deal with these was with respect to values. The National Institute of Clinical Excellence established a Citizens' Council to deliberate on value issues and published its own social value judgement paper (which went through a couple of editions) to try and sort the issue out. In turn, it was hoped that this would help its appraisal and guideline committees as they went about their work.^20^ The heart of the issue with respect to values and to many other problems emerging in appraisal and guideline committees was that empiricist principles enshrined in a fundamentalist view of EBM on their own are insufficient. At its most extreme, empiricism and prescriptive rule following leads to a position where it becomes impossible to say very much of practical use to clinicians or anybody else—other than the methods by which the original studies were done or the methods of systematic review of those studies, or both, are woefully inadequate, so more and better research is needed. More importantly, the evidence on its own, no matter technically how good, was not a black box out of which readymade answers came. A narrowly empiricist account of doing EMB implies that evidence will speak for itself and would not require interpretation. Yet evidence cannot speak for itself in this way^21^ It always requires interpretation.^22^ Making sense of the evidence and all the issues it generated required human judgement and inference.

The limitations and difficulties of the processes used by NICE at the time were explored by a number of members of the senior team at NICE as they wrestled with the difficulties involved.^23^, ^24^, ^25^, ^26^, ^27^, ^28^ Sir Michael Rawlins often made the point that guidelines were not tramlines and that factors and ideas beyond the empirical data were required both for sensible and sensitive clinical practice as well as for meaningful guideline development; but even at NICE where there was explicit acknowledgement of the difficulty, exactly how to integrate the ideas was never formally elaborated. Philosophically, the issues that NICE was struggling with had the empiricism‐rationalism distinction at their very heart. The effort to apply as rigorously as possible the precepts of EBM inevitably came up against the problems that the evidence was sometimes insufficient, that the evidence did not deal with the problem at issue, or that the evidence was equivocal. To make sense of it, interpretation and inference were required and judgements had to be made about the applicability, relevance, practicality, and acceptability of any recommendations arising from the empirical evidence. All of this required rationalist knowledge in conjunction with the empirical data. The textbooks and the manuals however for the most part deal only with handling the empirical information, not the rationalist knowledge.^28^

The knowledge and evidence to be found in the social sciences and humanities are particularly germane to this problem. Psychology, sociology, anthropology, political science, history, and philosophy all in various ways could make significant contributions. These disciplines describe and investigate human behaviour, individually, in groups and communities, in organizations, and in particular times and places. They constitute discrete evidence bases, which for the most part are never pressed into service by EBM. Psychology and Economics have made the greatest inroads but both within relatively narrow confines of health‐related behaviour change (psychology) and cost effectiveness and the application of cost utility theory (economics). Important as these contributions are, there is a very much wider knowledge base that could be used. Presently if this material appears at all, it is as a basis for describing context and social background, not as evidence that might be used to address questions of effectiveness or how to bring about change in complex systems. Taken as a whole, the world of EBM excludes much that, as is argued below, if a rationalist turn was taken, could help.

## 6 EMPIRICISM AND RATIONALISM

Empiricism is used here following Hume. In *An Enquiry Concerning Human Understanding*, Hume demarcated 2 types of knowledge, which he called rationalism and empiricism.^1^ Rationalism is for Hume based on demonstrative reasoning while empiricism is based on factual reasoning. Demonstrative reasoning is deductive. It proceeds from the general to the particular. It involves relationships between ideas. Demonstrative reasoning operates with complete certainty because it is based on the logical relations between ideas and concepts. Demonstrative reasoning is a priori, ie, it does not depend on experience or observation. It is pure theoretical knowledge. Relations between ideas—rational thinking—for Hume can be known a priori—before experience or observation.

Factual reasoning on the other hand for Hume is inductive and proceeds from the particular to the general, from observation and experience to more complex ideas. It involves drawing reasonable but not logically certain conclusions from empirical evidence, experience, or testimony. Factual reasoning is the basis of empiricism and is a posteriori, ie, after the fact of experience or observation. To learn from what we have observed, we must extrapolate beyond experience. To do this, we must draw out factual or inductive inferences from what we have observed to that, which we have not. Such inferences are contingent. This is because the future may not resemble the past and what we observe now or in the future may not be the same as we have observed in the past. For Hume, the relations between things, and especially causal relations, can only be known on the basis of experience. Factual reasoning, concerning the relation between things, is based on an assumption, used by all of us all as we go about our business in the world, which is the things we have observed in the past are a reasonable, but not infallible, guide to what we will observe in the future.

Matters of fact are not just about ideas and how they relate to each other but they are also about how things work in the real world. Hume concluded that rationalism and empiricism are fundamentally irreconcilable, and he opted for empiricism. *The Enquiry* ends with his famous words.

“If we take in our hand any volume of divinity or school of metaphysics, for instance; let us ask, Does it contain any abstract reasoning concerning quantity or number? No. Does it contain any experimental reasoning concerning matters of fact and existence? No. Cast it then into flames. For it can be nothing but sophistry and illusion.”^1^

The empiricism in Hume's sense is clear in the writings of Cochrane and Sackett, in the precepts of the Cochrane Collaboration and indeed in NICE's manuals. The empiricist position was clearly stated by Sackett and Rosenberg for example who argued that doctors should always seek to base their decisions on the best available evidence.^10^ They saw the problem as one of empirical information deficit. However, a number of practical, methodological, and philosophical problems emerge out of this view of things.

The rejection of rationalist thinking (in Hume's sense) exhibited by Sackett is more than a preference for particular methods; it is an epistemological position.^29^ In other words, it defines and privileges a way of knowing about the world. This has real consequences for what is considered admissible and inadmissible as evidence. The empiricist position privileges factual, observable, empirically derived information and data. However, there is something of a paradox; while the rhetoric of EBM is strongly empiricist, the precepts and procedures of EBM are a set of rationalist ideas about methods, bias, good scientific practice, and so on. Further, empiricism embraces knowledge derived from all scientific and social scientific disciplines from archaeology to anthropology and all points between. But clearly, this is not what the proponents of EBM had in mind. They meant a particular subclass of empirical information derived from the clinical sciences, collected using particular methods and tested and appraised using specific techniques, especially an exclusion criterion applying to almost all of the scientific canon as well as rationalist ideas more generally. At the same time, an inclusion criterion is applied to a subclass of knowledge, that derived from trials. In this context, Kemm noted that the heavy reliance on the RCT is particularly important. Randomized controlled trials produce in effect average results in what are often atypical populations (by virtue of the exclusion criteria applied to trial participants—aging and comorbidities, for example). Variations between members enrolled in the study are smoothed out in the overall result. Therefore, applying this result to an individual patient and determining particular patient treatment decisions always require clinical judgement rather than the simple application of the results of a trial or the results of meta‐analyses of trials. Patients are heterogeneous and so are the people enrolled in the trial. Subgroup analyses may help up to a point, but it does not solve the problem of patient variation.^2^, ^21^, ^30^ Other forms of knowledge, learning, and reasoning are necessary for clinical practice to deal with patient heterogeneity.

Further, not only are patients biologically heterogeneous they are also heterogeneous socially and psychologically. There are very important biological and social differences between individual patients that are concealed by the epistemological precepts of the RCT.^29^ There has been a tremendous effort aimed at maximizing internal validity and the relationship between intervention and outcome. The problem of external validity has received much less attention.^21^ This has had the unintended consequence of being a de facto barrier to information derived from sources other than RCTs. The degree to which factors that could be elicited from these other sources interact with biology is almost entirely absent from the way most trials are done with mostly social factors being used to control for confounding rather than being conceptualized as critical variables interacting on treatment outcomes and with the biology directly.^31^

Feinstein and Horwitz put it succinctly, arguing that EBM data

“do not include many types of treatments or patients seen in clinical practice; and the results show comparative efficacy of treatment for an ‘average’ randomized patient, not for pertinent subgroups formed by such cogent clinical features as severity of symptoms, illness, comorbidity, and other clinical nuances. The intention‐to‐treat analyses do not reflect important post‐randomization events leading to altered treatment; and the results seldom provide suitable background data when therapy is given prophylactically rather than remedially, or when therapeutic advantages are equivocal. Randomized trial information is also seldom available for issues in aetiology, diagnosis, and prognosis, and for clinical decisions that depend on pathophysiologic changes, psychosocial factors and support, personal preferences of patients, and strategies for giving comfort and reassurance.”^32^

In other words, there is knowledge both from other empirical approaches as well as rationalist ways of thinking that are quintessential to the clinical task, which in a narrow interpretation of EBM will be side‐lined.

Tonelli's criticisms of EBM echo this. He has argued that there are 5 types of consideration in making decisions about individual patients. These are as follows. Empirical evidence derived from clinical research. Experiential evidence derived from personal clinical experience or the clinical experience of others (ie, expert opinion). The pathophysiologic rationale based on underlying theories of physiology, disease, and healing. Patient values and preferences derived from personal interaction with individual patients. And system features including resource availability, societal and professional values, and legal and cultural concerns. Casuistic reasoning, he suggests, allows the physician to weight these different elements.^33^ In this latter regard, there is a wealth of data from the social sciences that is helpful, but which is potentially excluded from EBM a priori, unless steps are taken as for example NICE sought to do, to bring them into the frame.

Evidence‐based medicine has a tendency towards reductionism that derives from the centrality of the RCT. This strategy has important benefits. Biomedical reductionism has been very successful in helping to identify causal factors and in finding curative or palliative drugs and other interventions for many diseases. But because of biological variation between individuals, neither potentially causal factors nor pharmaceutical agents are universal in their causes and effects. Heterogeneity is endemic to medicine. Epidemiological studies and RCTs describe average effects in populations and subpopulations, and interpretation always has to be mindful of outliers consequent on heterogeneity. What the RCT does is reduce phenomena down to a unit of analysis in such a way that there can be clarity in the relationship (which is often an association rather than a mechanism)^34^ between 2 variables. Much of the effort at methodological refinement that has gone on over the years has been about getting better and better at reducing as far as possible the factors that might confound the relationship between the two variables. As an analytic strategy, this makes absolute sense. Practically, it is often helpful to reduce things down analytically, and science has proceeded down the years using precisely this strategy.

However, the analytic strategy is an abstraction from the real world, not the real world itself. The moment that the fact that it is an analytic strategy is lost sight of, problems will ensue. Much of the methodological literature about RCTs appears to reify the abstraction as an objective account of reality itself. In complex biosystems, in complex social systems, and in the complex relations between them, the notion that two variables act one on the other and on nothing else is clearly wrongheaded. Systems approaches draw attention to multiplex phenomenon with multiple interacting parts, which are in continuous and iterative and recursive relations. The models constructed of these are abstractions, not reality itself. So, the linearity implied in trials as well as their reductionism is not wrong in itself—understanding is advanced by using such strategies—but to shift the reduced linearity to the status of the real world is either deliberate or very naïve scientific legerdemain producing a tendency towards prescriptiveness.^35^

The hierarchy of evidence places meta‐analyses of well‐conducted RCTs at the top of the hierarchy and expert opinion, consensus, and case reports at the bottom. The hierarchy of *evidence* is a misnomer because it is a hierarchy of *methods* with the methods that are best at reducing bias between dependent and independent variables at the top.^2^ Abstractions are not empirical reality; they are ideas about, or representations of, reality not reality itself. They are in Humean terms rationalist ideas, and the hierarchy is an example of a rationalist construct—it is about relations between ideas about bias and their confounding effects on variables, which themselves only exist as analytic abstractions or as ideas. The hierarchy is not itself evidence based or empirical—it is an a priori judgement.^21^, ^28^

Evidence‐based medicine has a fundamental problem in dealing with the absence of evidence in two senses. First, when evidence exists, it is excluded on epistemic grounds—ie, it is excluded because it is not derived from RCTs or meta‐analyses of RCTs by invoking the hierarchy of evidence. What is happening here is that EBM is using judgements (rationalist thinking) either explicitly or implicitly from the very outset to determine what constitutes evidence in the first place. So contra the rhetoric, which makes claims about the importance of judgements in individual patient treatment decisions, judgements come into the process long before the patient encounter. Second, judgements come into play when the evidence does not exist. Evidence‐based medicine has a strong rhetoric about being evidence based, but often is not in its own terms when the evidence is not there. It faces the problem of what to do then. Sometimes, the argument is made that this is not actually a problem so long as the evidence has been searched for properly so what is unknown is really unknown^36^—but this is of course a rationalist principle not an empirical one, and searching is itself driven by a priori judgement criteria, which are rationalist rather than empiricist. Once again, great care has to be taken by guideline developers if they are not to fall into this trap.

Feinstein and Horwitz note that

“To obtain ‘trustworthy’ information, randomized trials have concentrated on getting ‘hard’ data about death, disease, and demography. The patient's baseline condition, before therapy, is regularly characterized with the ‘reliable’ information of age, gender, race, imaging, endoscopy, biopsy, cytology, and laboratory tests. The therapeutic outcome is cited, whenever pertinent, as death, or in global ratings for certain symptoms (pain, insomnia, etc.) that need not be specified more precisely because the ‘double‐blind’ observations will presumably avoid bias”.^32^

Therefore, the belief that randomization solves problems of heterogeneity is misplaced.

There is one further important point to note about the idea of chance and statistical significance in assessing trial results. This is also a theoretical principle. Specifically, the idea is about reducing the likelihood that what has been observed is the consequence of chance rather than the hypothesized relationship which is being investigated. However, actually nothing happens by chance; all things have preceding causes. When we say it happened by chance, it really means we did not know in advance that there was some alternative cause for what had happened to that which we are investigating or have hypothesized. To argue that this helps to overcome the fact that we do not know in advance all possible reasons for things happening is a logical principle. It is not an empirical one. It is a helpful principle when it comes to interpreting statistical data, but a rationalist principle it remains. To say something may have happened by chance is a rationalist theoretical construct not an empirical one grounded in evidence.

And the final irony is this. The orthodoxy of EBM belongs firmly in the rationalist camp. It is an overarching set of theoretical ideas and logical principles about methods. The structure of the design of the RCT and the hierarchy of evidence are prime examples as are certain epidemiological and statistical techniques; the hierarchy of evidence and the associated idea of internal validity are rationalist constructs not empirical ones. The principles of trial design and internal validity upon which it is based are also rationalist ideas. This means that they are derived from a priori theoretical and logical principles rather than a posteriori observations. These things may give the impression of being about hard empirical science, when in fact they are rationalist a priori constructs. A strict application of the principles of EBM will drive out rationalist knowledge, an anxiety echoed early on by Tonelli.^29^ The problem or the bind here is that once other forms of evidence or ways of thinking are acknowledged to be important and legitimate, the concept of best available evidence is undermined.^33^

## 7 CONCLUSION

However, we must be careful not to throw the baby out with the bathwater. The fact is that despite the overarching tendency to emphasize a particular version of empiricism, EBM and its aspirations are worthy and worthwhile, and the move to placing evidence at the heart of decision making is certainly one that deserves support. The difficulty arises with the knotty problem of judgement and other forms of rationalist knowledge, which while they have been acknowledged from the very beginning of EBM still appear to sit uncomfortably with the empiricist orthodoxy. Others have considered this problem. Jenicek argued for a new type of science to help to find a way through the difficulties.^37^ Loughlin et al took issue with this solution and highlighted the fundamental irreconcilability of rationalism and empiricism.^38^ While the ideas of rationalism and empiricism are philosophically apparently irreconcilable, in practice in organizations like NICE, for example, guidelines de facto can only be developed if both types of knowledge are deployed, in a process akin to using Kant's analytic and synthetic judgements.^27^, ^28^

The solution lies in rectifying the enormous imbalance between the amount of effort that has gone into refining certain methods like the trial, and the associated statistics and epidemiology on the one hand and the forms of reasoning so often consigned to the bottom of the evidence hierarchy on the other. There are well‐defined scientific protocols for methods and interpretation of results. The methods for understanding processes of inference and judgement beyond the method protocols are less well understood or articulated, and they should be developed.

As a result, enormous effort has also gone into refining certain but not all aspects of the competencies involved, and a huge amount of development in the infrastructure to support evidence cumulation and synthesis. Judgement and interpretation as part of the practice have been set to one side in favour of a highly empiricist account of the activity. Kathryn Montgomery has plausibly argued that to understand medicine, we must get behind its rhetoric to the actual practice of medicine.^3^ Taking our cue from this, we need to get behind the overly simplistic rhetoric of EBM and see it as a practice that works with complex problems all the time. Practices require practical reasoning, in the case of medicine as Montgomery argued that practical reasoning is the combination of scientific information, clinical skill, and experience. In Aristotelian terms, this is phronesis. Phronesis goes beyond analytical scientific knowledge and technical knowledge or know how and involves judgement.^39^ The task is to describe that process of phronesis and to understand any cognitive biases inherent in our judgement processes. What we need to do is set out a new programmatic statement, which delimits all the problem areas that have been identified in this essay as rationalist thought, so not just judgement, but decision‐making, causation, reductionism, and values for example. Our extant knowledge of the sociology of science should be pressed to service to see where philosophical ideas along with other evidence drawn from the social sciences will help to specify and articulate the problems.

In the same way that several generations ago Archie Cochrane and David Sackett mapped the problems and set in motion an agenda that has served us well, we must now try to do the same for the bits that have not been discussed anywhere nearly so precisely. We also need to make plain that this will not in some sense diminish EBM—it is not a retreat from science back to anecdote. Rather, if EBM is to progress, it will have to get to grips with a set of problems, which if forever consigned to the status of non or soft knowledge will seriously hinder further scientific progress.
